# Supplementary material for: The sterol C-24 methyltransferase encoding gene, erg6, is essential for viability of Aspergillus species
Source: Nat Commun. 2024 May 20;15:4261. doi: 10.1038/s41467-024-48767-3 (PMC11106247; doi:10.1038/s41467-024-48767-3)
Supplement: Supplementary file 1 — Supplementary Information [file 41467_2024_48767_MOESM1_ESM.pdf]

A.

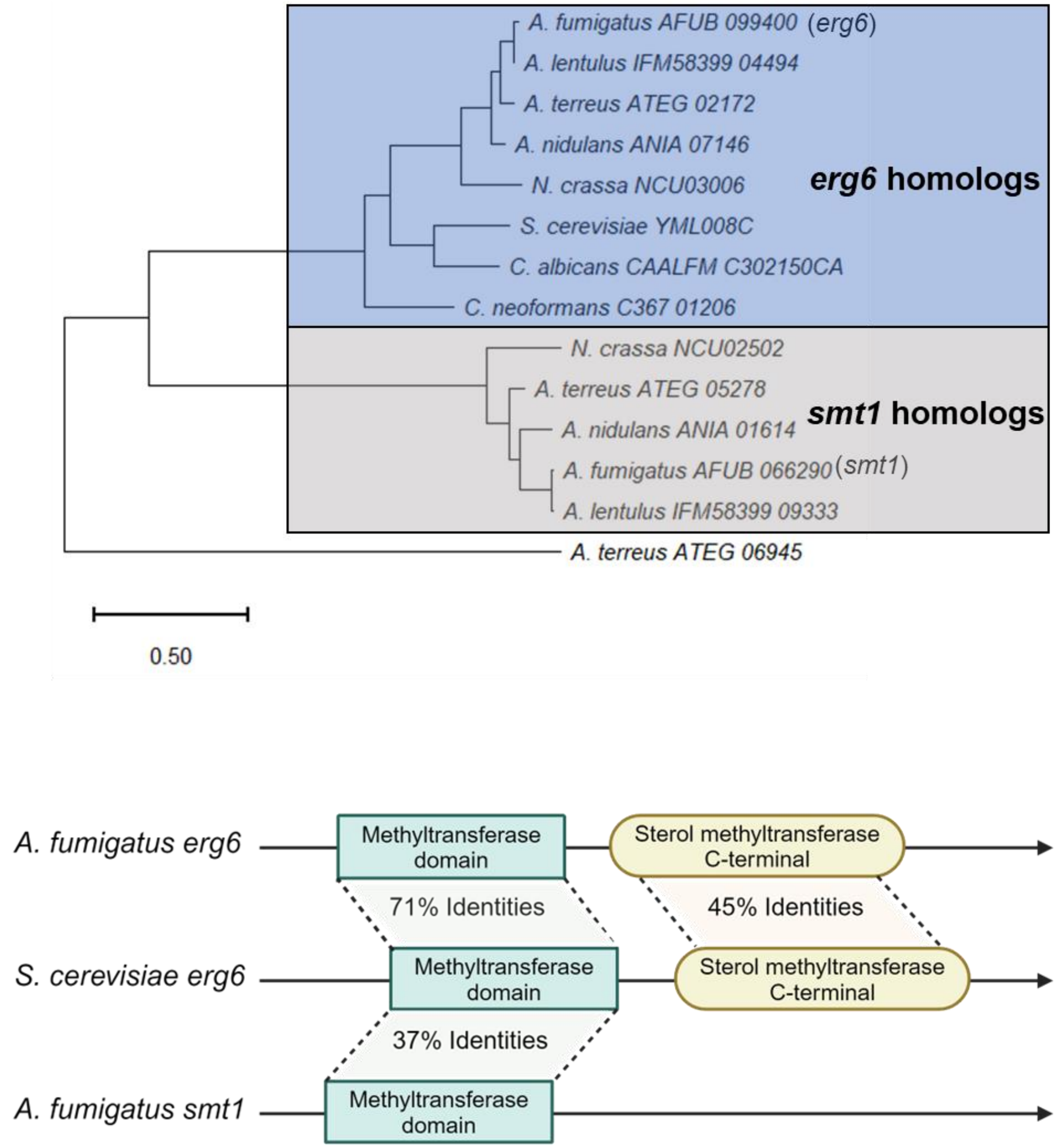

B.

Figure S1. Comparison of Erg6 protein similarities among select fungal species. (A) Phylogenetic analysis of the homologs of sterol C-24 methyltransferase encoding genes from selected model and pathogenic fungi. The putative full-length amino acid sequence of each organism was acquired by BLASTP analysis using the *erg6* of *S. cerevisiae* (SGD: S000004467) as a query sequence against the indicated database. Alignment analysis was performed by CLUSTALW and a phylogenetic tree was constructed by MEGA 11 software using the maximum likelihood method with a bootstrap value of 1000. Organisms used for comparison are *A. fumigatus*, *A. lentulus*, *A. terreus*, *A. nidulans*, *Neurospora crassa*, *S. cerevisiae, C. albicans* and *C. neoformans*. **(B)** The conserved domain of two putative sterol C-24 methyltransferase proteins of *A. fumigatus* and *S. cerevisiae* Erg6p were predicted by the NCBI conserved domain database (https://www.ncbi.nlm.nih.gov/Structure/cdd/wrpsb.cgi). The identities of the predicted protein domains compared to the query sequence (*S. cerevisiae* Erg6p) are indicated in the illustration.

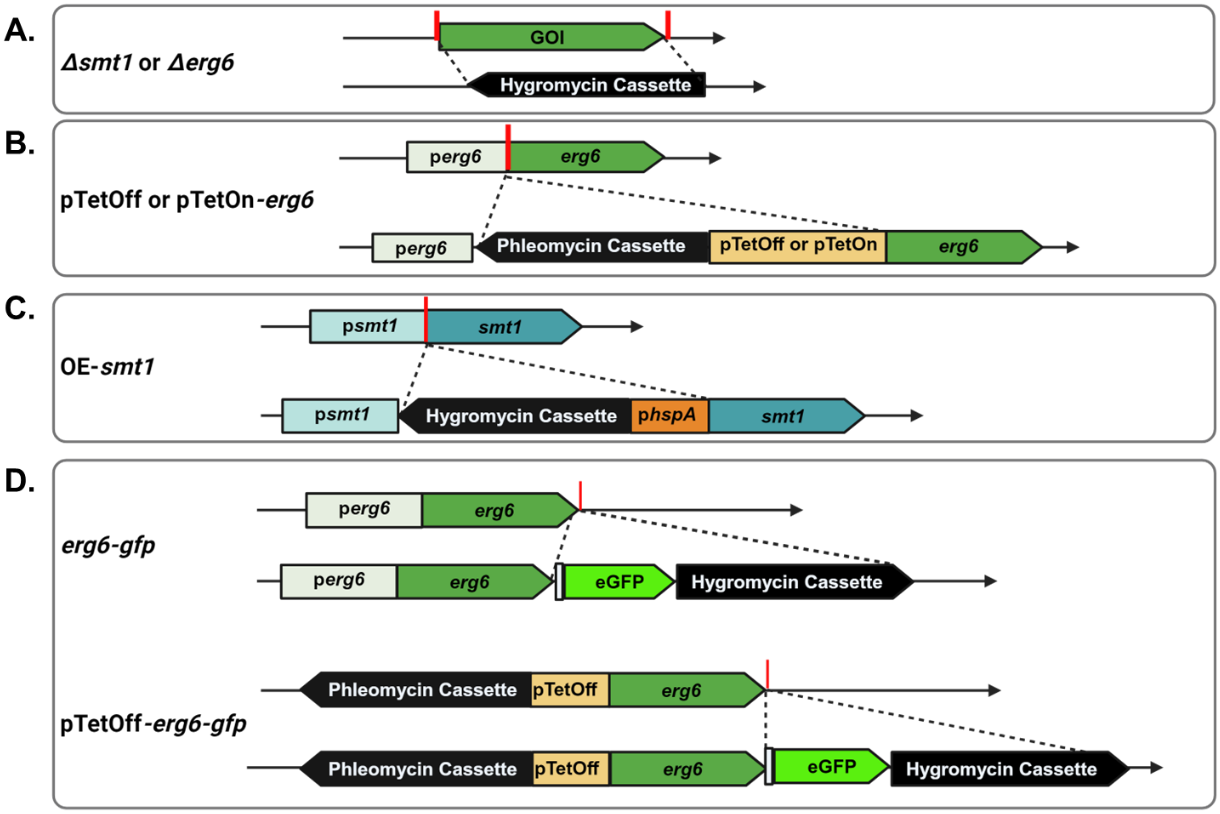

Figure S2. Schematic of gene manipulations by CRISPR/Cas9 editing. (A) Deletion of the gene of interest (GOI). Two protospacer adjacent motifs (PAMs, indicated as red bars) flanking the GOI were targeted by repair templates composed of a hygromycin resistance cassette with ~40-basepair microhomology regions for integration upstream and downstream of the GOI in the Δ*akuB*⁻ pyrG+ genetic background to generate Δ*smt1* and Δ*erg6* mutant. **(B)** Tetracycline repressible or inducible expression and overexpression (OE) **(C)** of GOI. A PAM site upstream of the GOI was targeted with a repair template carrying the TetOff or TetOn promoter construct or the strong pHspA promoter fused to either a phleomycin or hygromycin resistance cassette. **(D)** Generation of GFP-tagged Erg6. A repair template containing a linker sequence, the egfp coding sequence, and a phleomycin resistance cassette was amplified using primers to incorporate microhomology regions on either side of a PAM site selected at the 3' end of *erg6*.

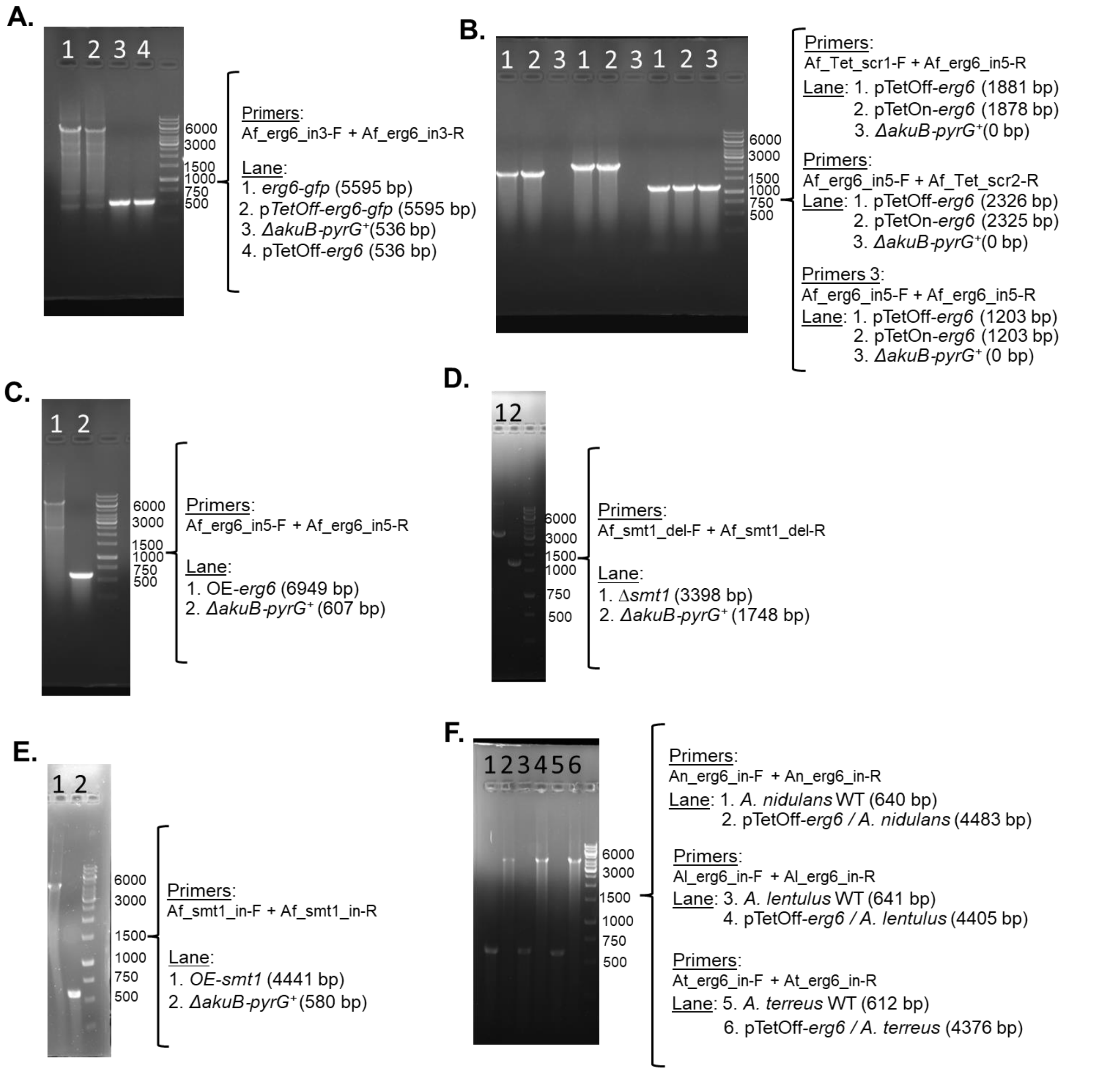

Figure S3. Agarose gel electrophoresis of polymerase chain reaction (PCR) screening for the mutants constructed in this study. (A) Confirmation of erg6-gfp and pTetOff-erg6-gfp strain construction. **(B)** Confirmation of pTetOff and pTetOn integration upstream of *A. fumigatus erg6*. **(C)** Confirmation of HspA promoter integration upstream of *A. fumigatus erg6* to construct the *erg6* overexpression (OE) mutant. **(D)** Confirmation of *A. fumigatus smt1* gene deletion. **(E)** Confirmation of HspA promoter integration upstream of *A. fumigatus smt1* to construct the *smt1* overexpression (OE) mutant. **(F)** Confirmation of pTetOff integration upstream of *erg6* in *A. nidulans*, *A. lentulus*, and *A. terreus*. Primers are listed in Supplemental Table 2.

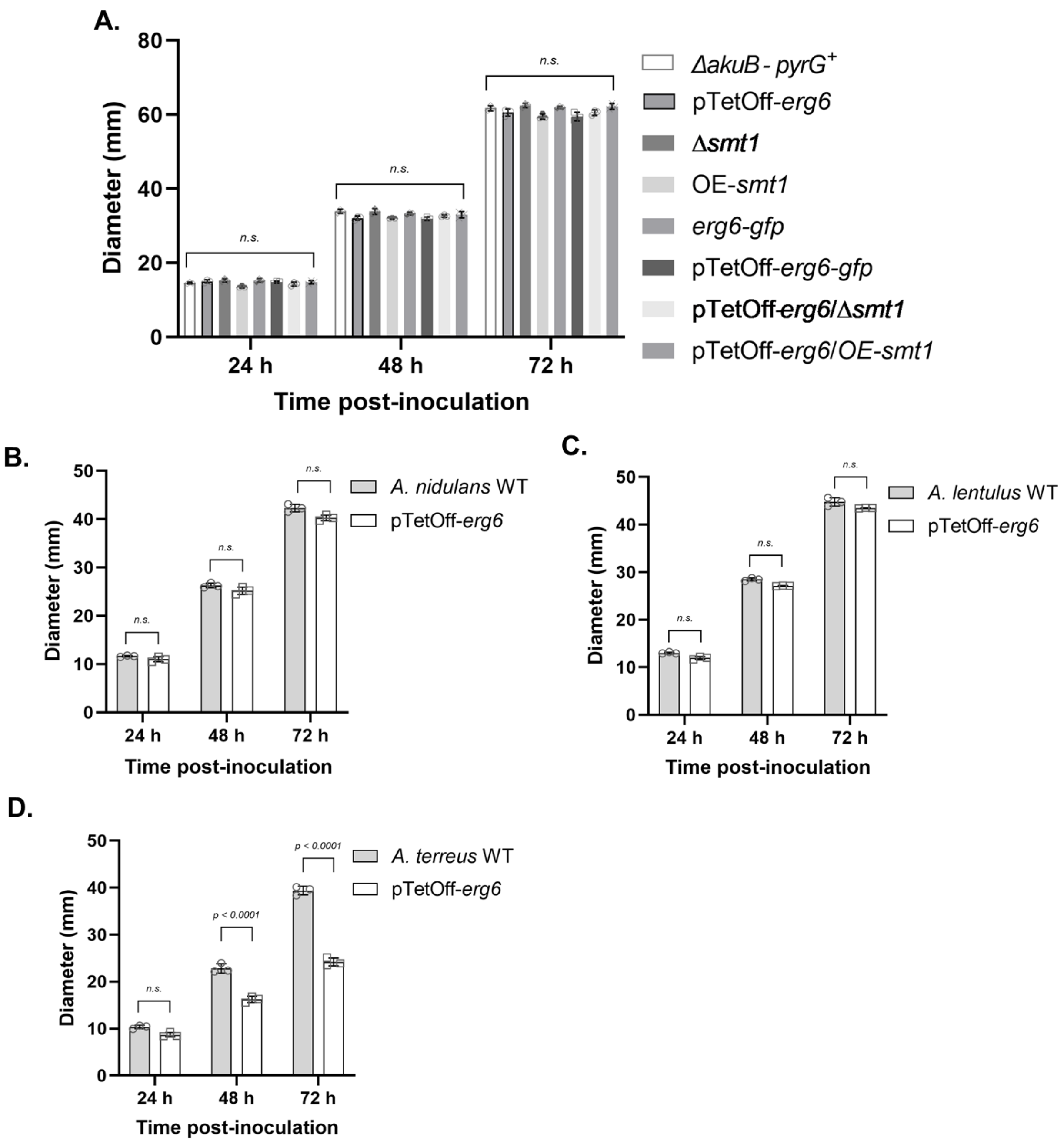

Figure S4. Comparison of colony diameters among mutant strains. Colony diameter was recorded for each day post-inoculation for mutant strains constructed in **(A)** *A. fumigatus*, **(B)** *A. nidulans*, **(C)** *A. lentulus*, and **(D)** *A. terreus*. Colonies were grown from 10,000 conidia spot-inoculated onto GMM agar at 37°C. Data are presented as mean ± SD and compared using 2-way ANOVA. ns = not significant.

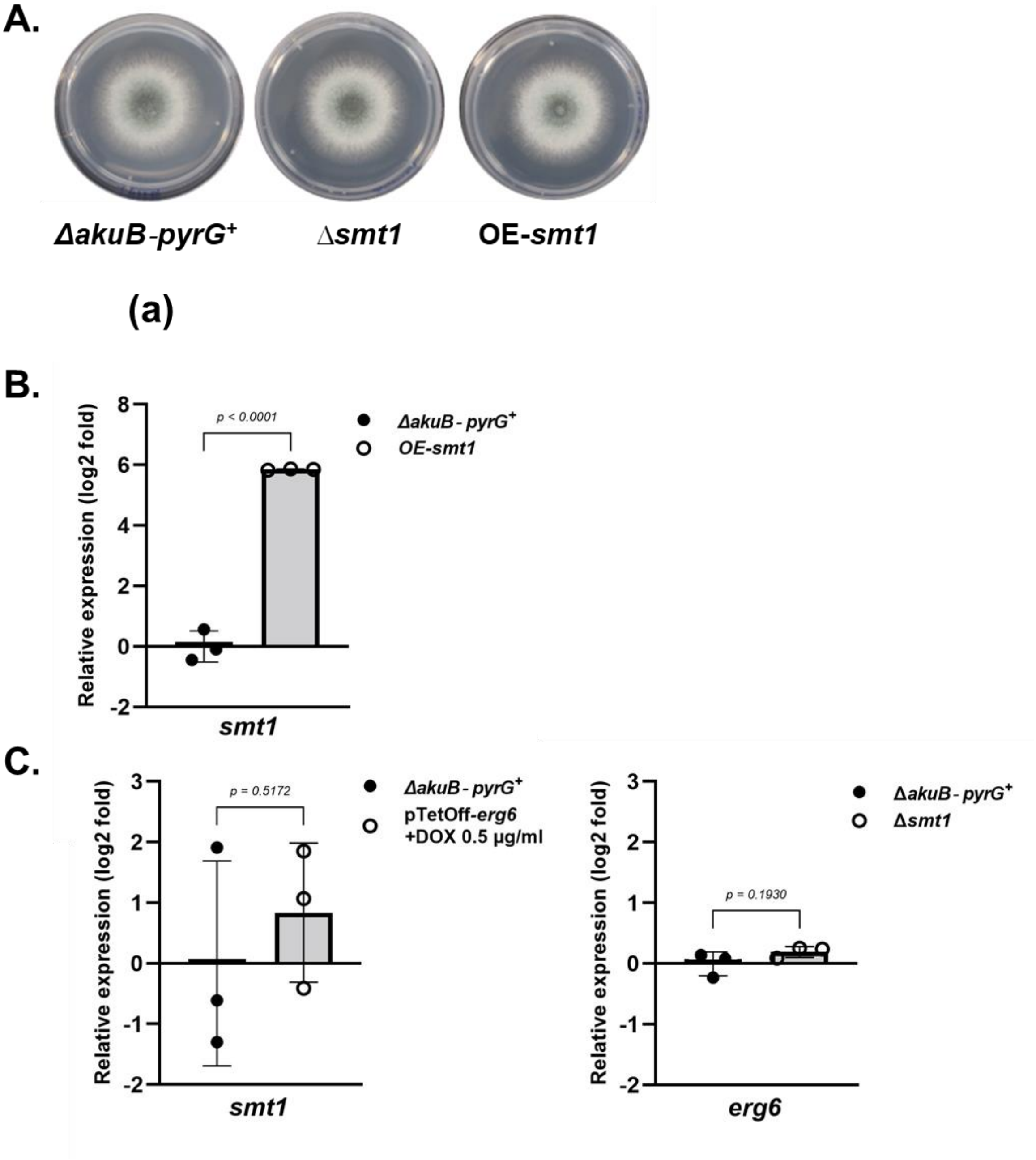

Figure S5. Analyses of the putative *A. fumigatus erg6* paralog, *smt1*. (A) Colony morphology of the parental strain, Δ*smt1*, and OE-*smt1*. A total of 10,000 conidia were inoculated onto GMM agar plates and incubated for 48 h at 37°C. **(B)** The expression level, as measured by RT-qPCR, of *smt1* after promoter replacement mutation using the pHspA promoter. **(C)** Expression changes in *smt1* and *erg6* in response to loss of the respective paralogs. Mycelia were harvested after 16 h in liquid GMM at 37°C/250 rpm. Gene expression was normalized to the reference gene, *tubA*, and data presented relative to parental strain as mean ± SD of $\log_2$ fold change. n = 3 independent experiments. Two-tailed Student t-test was used for statistical analysis.

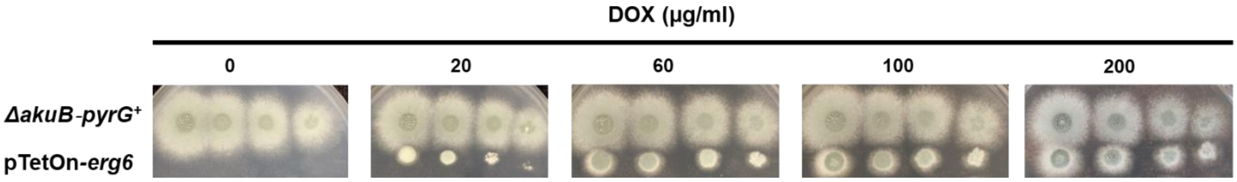

Figure S6. Growth of the pTetOn-*erg6* mutant is doxycycline dependent. Spot-dilution assays of the parental and pTetOn-*erg6* strains were performed on GMM agar plates using the indicated doxycycline levels. Culture conditions were as described in Fig. 1B. DOX = doxycycline.

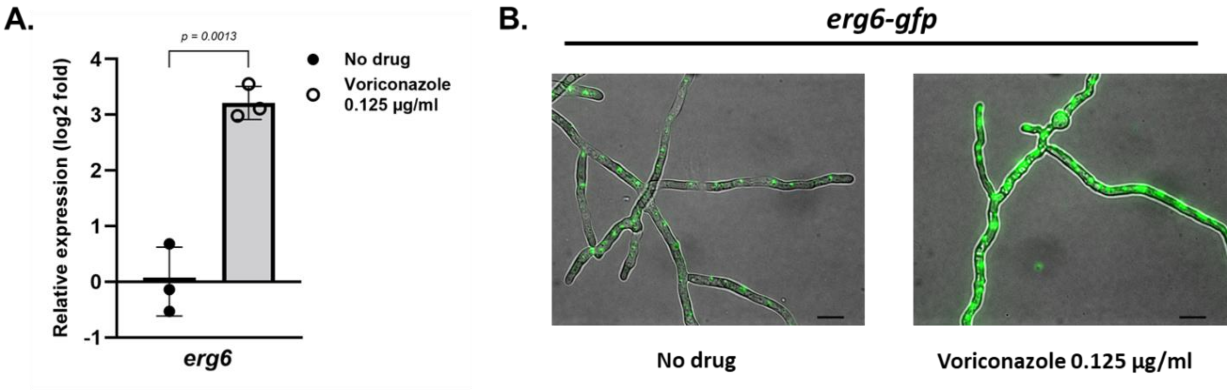

Figure S7. Voriconazole treatment increases *erg6* expression but does not alter protein localization. (A) RT-qPCR analysis of *erg6* expression with or without voriconazole treatment. Mycelia were harvested after 16 h in liquid GMM supplemented with or without 0.125 µg/ml voriconazole at 37°C/250 rpm. Gene expression was normalized to the reference gene, *tubA*, and data is presented relative to control group as mean ± SD of log_2 fold change. n = 3 independent experiments. Two-tailed Student t-test was used for statistical analysis. **(B)** Mycelia were cultured in GMM broth with 0.125 µg/ml voriconazole for 16 h at 37°C. Fluorescent images were captured using GFP filter settings. Scale bar=10 µm.

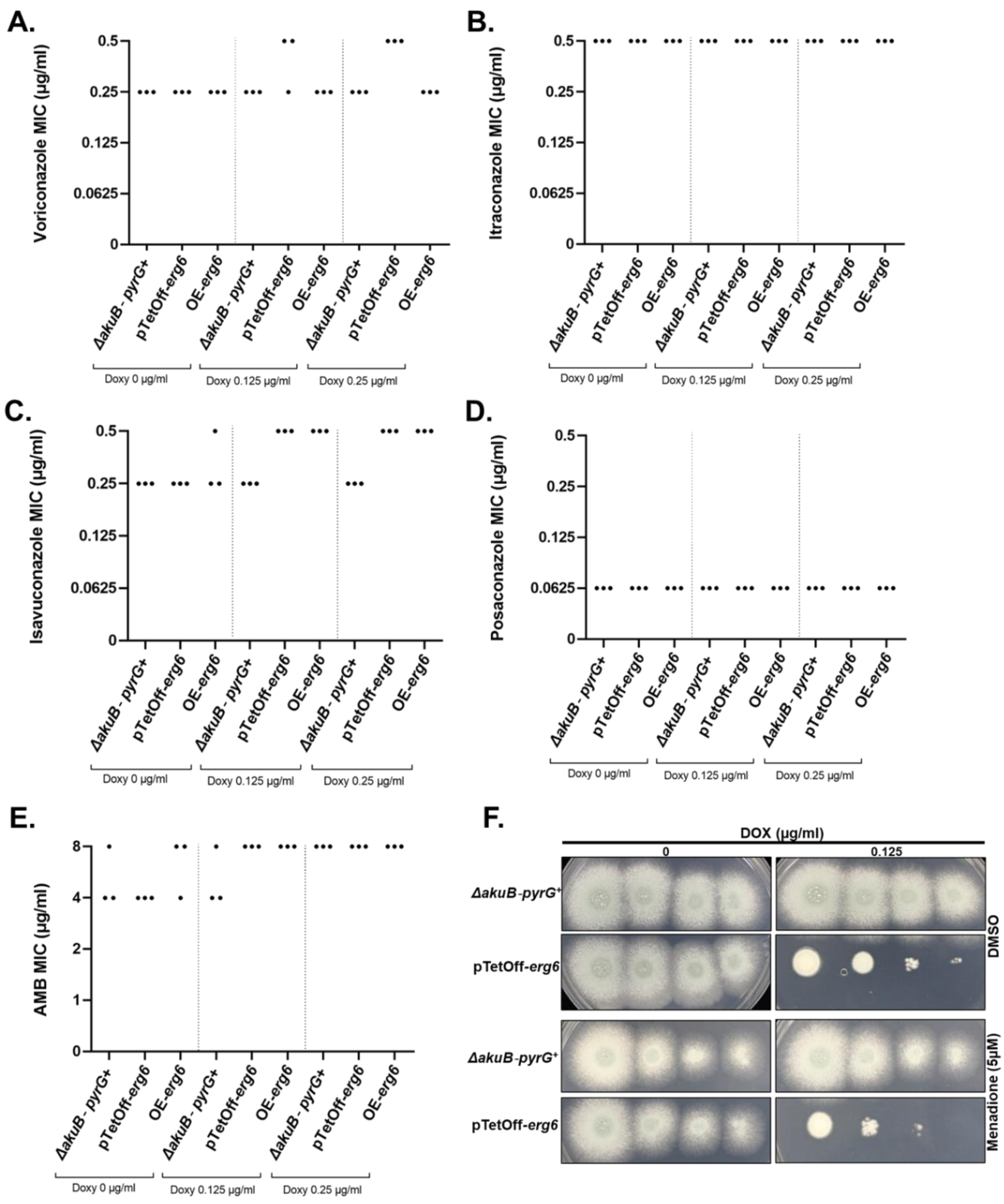

Figure S8. Repression of *erg6* does not alter antifungal susceptibility profiles in *A. fumigatus*. Broth dilution antifungal susceptibility assays for **(A)** voriconazole, **(B)** itraconazole, **(C)** isavuconazole, **(D)** posaconazole, and **(E)** amphotericin B (AMB) were performed in triplicate for each strain using the indicated doxycycline concentration. Assays were conducted according to the CLSI standard M38-A2. **(F)** Repression of *erg6* expression does not result in resistance to menadione stress. Spot-dilution assays were performed as described in Figure 1B in the presence or absence of the indicated concentration of menadione or vehicle control (DMSO). DOX = doxycycline.

Supplementary Table 1. Strains used in this study.

Species	Strain	Genetic background	Source
A. fumigatus	Δ*akuB-pyrG*$^{+}$	KU80Δ*pyrG*	Ref. 1
	Δ*smt1*	Δ*akuB-pyrG*$^{+}$	This study
	pTetOff-*erg6*	Δ*akuB-pyrG*$^{+}$	This study
	pTetOn-*erg6*	Δ*akuB-pyrG*$^{+}$	This study
	OE-*smt1*	Δ*akuB-pyrG*$^{+}$	This study
	pTetOff-*erg6* /OE-*smt1*	OE-*smt1*	This study
	pTetOff-*erg6* / Δ*smt1*	Δ*smt1*	This study
	erg6-gfp	Δ*akuB-pyrG*$^{+}$	This study
	pTetOff-*erg6*-gfp	pTetOff-*erg6*	This study
A. lentulus	DI-19-116	-	Gift from Nathan P. Wiederhold, PharmD
	pTetOff-erg6 / DI-19-116	DI-19-116	This study
A. terreus	FGSC A1156	-	Fungal Genetics Stock Center
	pTetOff-*erg6*/ FGSC A1156	FGSC A1156	This study
A. nidulans	FGSC A1166	-	Fungal Genetics Stock Center
	pTetOff-*erg6*/ FGSC A1166	FGSC A1166	This study

Supplementary Table 2. Primers in this study.

Purpose	Primer name	Sequence
Protospacer Sequence	Af_Cr_erg6_5’	AAGTCCAATTGCTATCGCCA
Protospacer Sequence	Af_Cr_erg6_3’	TGTAAAAGAGACTCGTTACT
Protospacer Sequence	Af_Cr_smt1_5’	TGTGGGCGGTGATAGCGGAC
Protospacer Sequence	Af_Cr_smt1_3’	TCATTGCTCAAAAGCCTTCG
Protospacer Sequence	At_Cr_erg6_5’	ACCGCCACCCGTTCTCGCCA
Protospacer Sequence	An_Cr_erg6_5’	ACTGTCAATTCAATTCACAA
Protospacer Sequence	Al_Cr_erg6_5’	AAGTCCAATTGCTATCGCCA
Repair Template	Af_erg6_del RT-F	GATCTGTGATCCACCCCTTTTCCACCCTTACCACATCCAAAGCTT GCATGCCTGCAGG
Repair Template	Af_erg6_del RT-R	AGGATGCAGGCACAGGGCAGATATTTGTACAGGCAATTCGCCG AGCTCCCAAATCTGTCCAG
Repair Template	Af_erg6_OE RT-F	CTGATCTGTGATCCACCCCTTTTCCACCCTTACCACATCCATTCG TTGACCTAGCTGATTCTGG
Repair Template	Af_erg6_OE RT-R	GCGCAAGTGGTTCTCTTGTTCCAAAGCTACGGGGGCCATGGGG ATCGAATTCCTGCAGCC
Repair Template	Af_smt1_del RT-F	AGCATATCTCAACCTGCCTTGGAATCCAGCTTCTCTTTCTAGCTT GCATGCCTGCAGG
Repair Template	Af_smt1_del RT-R	CCAATTGCTCTTGTTTTTTGAGCGGGTGGTTAACAACTGACCGA GCTCCCAAATCTGTCCAG
Repair Template	Af_smt1_OE RT-F	AGCATATCTCAACCTGCCTTGGAATCCAGCTTCTCTTTCTAGCTT GCATGCCTGCAGG
Repair Template	Af_smt1_OE RT-R	GCCAGGGCCGGTGCTGTGGTCTGTGTTTCCATCATTGTTGTGTG AAGAAGTGAGGAGGGTTTCGT
Repair Template	Af_erg6_TetOff RT-F	GATCTGTGATCCACCCCTTTTCCACCCTTACCACATCCAACAATT AAAGCCTTCGAGCGTCC
Repair Template	Af_erg6_TetOff RT-R	CGCGCAAGTGGTTCTCTTGTTCCAAAGCTACGGGGGCCATCCG GTGATGTCTGCTCAAGC
Repair Template	At_erg6_TetOff RT-F	AATTCCGTCCACCCTCATTCCTCCCCCTTTTTTACCCAGAATTA AAGCCTTCGAGCGTCCC
Repair Template	At_erg6_TetOff RT-R	CACGAGAGTGGTCCTCGCGTTCGAGAGCGGTGGGAGCCATCC GGTGATGTCTGCTCAAGC
Repair Template	An_erg6_TetOff RT-F	CTTTCGCCTCCCCCCTTTTCAACCTCCTACTTTTCGATTCAATTA AAGCCTTCGAGCGTCCC
Repair Template	An_erg6_TetOff RT-R	CGCGCTGGTGGTTCTCCTTCTCTAAAGCAGTGGGAGCCATCCG GTGATGTCTGCTCAAGC
Repair Template	Al_erg6_TetOff RT-F	GATCTGTGATCCACCCCTTTTCCACCCTTACCACGTCCAAAATTA AAGCCTTCGAGCGTCCC
Repair Template	Al_erg6_TetOff RT-R	CGCGCGCGTGATTCTCCTGTTCCAAAGTAGCAGGGGCCATCCG GTGATGTCTGCTCAAGC
Repair Template	Af_erg6_TetOn RT-F	GATCTGTGATCCACCCCTTTTCCACCCTTACCACATCCAAAATTA AAGCCTTCGAGCGTCCC
Repair Template	Af_erg6_TetOn RT-R	CGCGCAAGTGGTTCTCTTGTTCCAAAGCTACGGGGGCCATGTG ATGTCTGCTCAAGCGGG
Repair Template	Af_erg6_GFP RT-F	CTTCACGCCCATGTATTTGATGGTCGGACGCAAGCCCGAGAGAT CTGGATGCGGCCGCATGGTGAGCAAGGGCGAGGA
Repair Template	Af_erg6_GFP RT-R	GGCAGATATTTGTACAGGCAATTCGTGTAAAAGAGACTCGAGCT TGCATGCCTGCAGG
PCR screening	Af_erg6_in5-F	GGCAGAATGGTCAGGTACTGC
PCR screening	Af_erg6_in5-R	CAATGTGGCATACTCAGCACGG
PCR screening	Af_erg6_in3-F	GCCGGATCGTTCAAGCACATG

PCR screening	Af_erg6_in3-R	GCCAATTACATAGGCAGGTCTCATG
PCR screening	Af_smt1_in-F	ACCCATGGCAGGTCATATGGC
PCR screening	Af_smt1_in-R	CTCGTCCGACCAGTCAAGGT
PCR screening	Af_smt1_del-F	GCAGGTCATATGGCGATTATCCG
PCR screening	Af_smt1_del-R	CTGTGAGTCGTGACACGTGC
PCR screening	At_erg6_in-F	CGTCTCAATTGCTTGCTGCC
PCR screening	At_erg6_in-R	AGCCGACATCCAGCACCTTC
PCR screening	An_erg6_in-F	GCGGTGTCGTGATTGTACAATCG
PCR screening	An_erg6_in-R	CCAACATCGAGCACCTTCATGC
PCR screening	Al_erg6_in-F	TGCTTCAAGATTGTGATCCTGTGG
PCR screening	Al_erg6_in-R	TGGTGAGCCAGGTAGTGTTCATG
PCR screening	Af_Tet_scr1-F	CACAACTAGACACTCAAGACGCG
PCR screening	Af_Tet_scr2-R	CGTCTACCAGTAGCCAGGAGTC
qRT-PCR	Af_erg6-qPCR-F	ACCCGACACTATTACAACCTGGC
qRT-PCR	Af_erg6-qPCR-R	GGTGAGCCAGGTAGTGTTCATGAC
qRT-PCR	Af_smt1-qPCR-F	TCTGGTGAAGGTTCACGCACTG
qRT-PCR	Af_smt1-qPCR-R	GAGCAATGATGCGGTAACTCGTAC
qRT-PCR	Af_abcC-qPCR-F	CTGGAGAAGGTCTCAATGTCGAAC
qRT-PCR	Af_abcC-qPCR-R	TTGGCCGTGCTTGGTAAGAG
qRT-PCR	Af_mdr1-qPCR-F	TCGTTATGTCACTCCCTGAGGG
qRT-PCR	Af_mdr1-qPCR-R	GATTCGGAGTCAAGAGCAGATGTG

SUPPLEMENTARY REFERENCES

1. Al Abdallah Q, Martin-Vicente A, Souza ACO, Ge W, and Fortwendel JR. C-terminus proteolysis and palmitoylation cooperate for optimal plasma membrane localization of RasA in *Aspergillus fumigatus*. *Front Microbiol* **9**, 562 (2018).
